# A New Case of Severe Allergic Contact Dermatitis Following a Recreational Exposure to Epoxy Resin

**DOI:** 10.1111/cod.70229

**Published:** 2026-08-03

**Authors:** Eva Ibeka, Olivier Dereure, Nadia Raison‐Peyron

**Affiliations:** ^1^ Department of Dermatology University of Montpellier Montpellier France

**Keywords:** allergic contact dermatitis, CAS 25068‐38‐6, case report, epoxy resin, facial dermatitis, recreational

The increasing popularity of creative leisure activities has resulted in the emergence of non‐occupational allergic contact dermatitis (ACD) to epoxy resins, and we report here a new case.

## 1 Case Report

A 41‐year‐old female physiotherapist with a history of atopic dermatitis and ACD to fragrances previously confirmed by positive patch tests presented with a severe facial dermatitis following the first use of an epoxy resin system purchased on line for home jewellery making without using personal protective equipment. The product label of the epoxy resin (bottle A) mentioned the presence of bisphenol A diglycidyl ether (CAS 25068‐38‐6) and alkyl (C12‐C14) diglycidyl ether (CAS 68609‐97‐2). The hardener (bottle B) contained benzyl alcohol (CAS 100‐51‐6), amines such as polypropylene glycol diamine (CAS 9046‐10‐0), isophorone diamine (CAS 2855‐13‐2) and 1,3‐cyclohexanedimethanamine (CAS 2579‐20‐6).

Within 6 h of exposure, the patient developed an itchy erythematous, edematous eruption involving the face, neck and upper chest (Figure 1). Owing to the aspect and location of the rash, airborne ACD was primarily suspected. Treatment with dexchlorpheniramine and oral corticosteroids led to resolution of symptoms in several days with residual xerosis.

**FIGURE 1:**
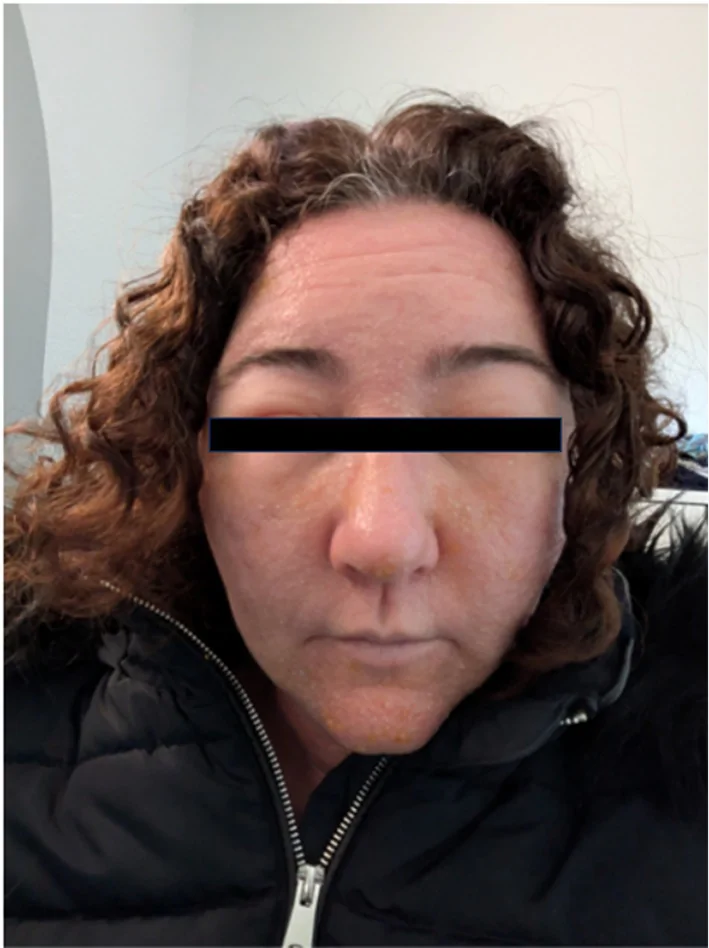
Acute erythematous, edematous and vesicular dermatitis of the face.

Several months earlier, she had bought in a discount store acrylic resins in powder and liquid form designed to be mixed together for creative hobby activities and used it with no problem whatsoever. She didn't wear artificial nails.

Patch testing was performed with the European baseline series, epoxy series, acrylates series and fragrance series (Chemotechnique Diagnostics, Vellinge, Sweden) except the 1,4‐butanediol diglycidyl ether (1,4‐BDDE), which comes from Allergeaze (SmartPractice Canada). The epoxy resin and its hardener were tested using 0.1% pet semi‐open tests. Readings were performed on day (D)2 and D3 according to ESCD guidelines, with a late reading at D7 with photos if necessary [1].

Positive reactions were observed for (Figure 2):

− Epoxy resin, bisphenol A (1% pet) ++ on D2 and +++ on D3 with current relevance and fragrance mix I (8% pet) ++ on D2 and D3 (past relevance) from the baseline series,
− Epoxy resin, bisphenol F (0.25% pet) ++ on D2 and +++ on D3, 1,4‐BDDE (0.25% pet) ++ on D2 and D3 from the epoxy series,
− Bisphenol A glycerolate dimethacrylate (bis‐GMA) (2% pet) and 2,2‐bis(4‐(2‐methacryl‐oxyethoxy)phenyl)propane (bis‐EMA) (2% pet) + on D2 and ++ on D3 from the acrylate series,
− Isoeugenol (2% pet) + on D2 and D3 with past relevance, from the fragrance series,
− Bottle A 0.1% pet: +/− on D2 and ++ on D3.

**FIGURE 2:**
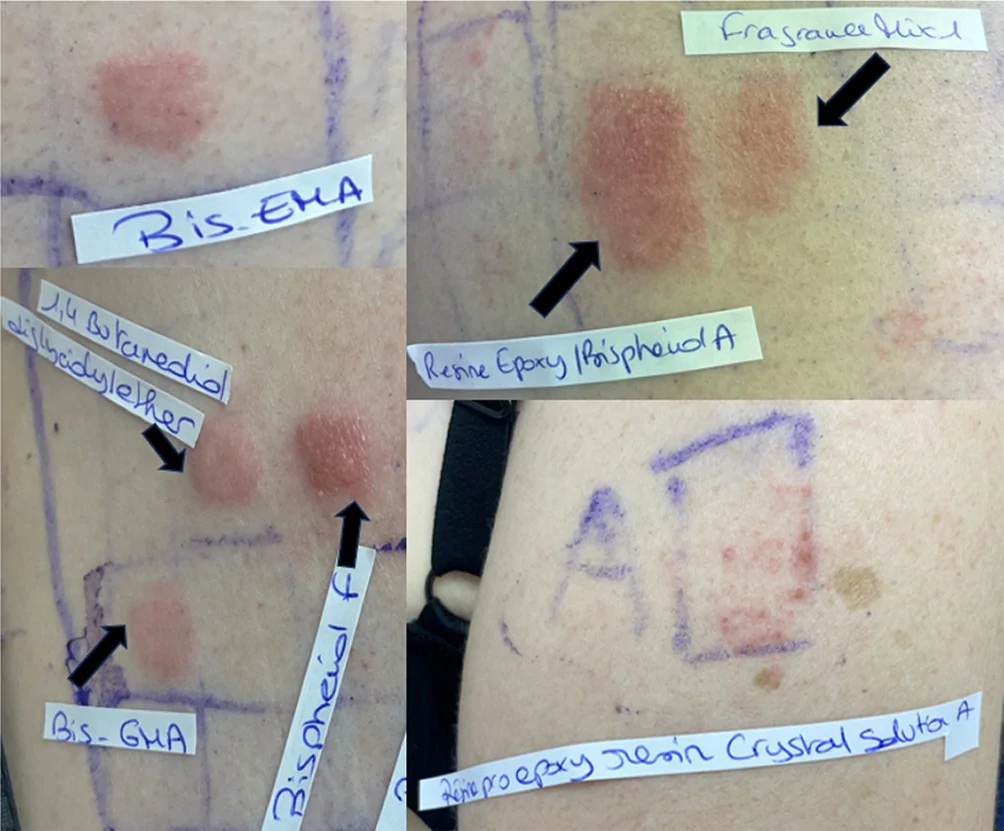
Positive patch tests on D3: Epoxy resin bisphenol A +++, fragrance mix I ++, 1,4 BDDE ++, bis‐EMA ++, bis‐GMA ++, bisphenol F +++, bottle A ++.

## 2 Discussion

Epoxy resin systems are associations of epoxy resins, curing agents (hardeners) and modifiers [2]. Most epoxy resin systems contain epoxy resins based on diglycidyl ether of bisphenol A (epoxy resin, bisphenol A) and/or based on diglycidyl ether of bisphenol F (epoxy resin, bisphenol F) [2]. These chemicals are well‐known sensitizers, and patients sensitized to diglycidyl ether of bisphenol F (DGEBF)‐based resins often equally react to diglycidyl ether of bisphenol A (DGEBA)‐based ones [3, 4]. DGEBA‐R is the most common sensitizer in epoxy resin systems [5].

Our patient was also sensitized to 1,4‐BDDE which is an epoxy reactive diluent. The bottle A contained alkyl (C12‐C14) diglycidyl ether which hypothetically cross‐reacts with 1,4‐BDDE.

The positive reactions to the epoxy acrylates bis‐GMA and to bis‐EMA may be explained by possible cross‐allergy between DGEBA and epoxy acrylates [6]. The rapid allergic reaction after epoxy resin contact may be due to prior exposure to epoxy acrylates in a two‐component acrylate product of unknown composition used by the patient.

Epoxy compounds represent one of the most frequent sensitizers responsible for occupational contact dermatitis in Europe [5]. However, recent reports highlight the emergence of non‐occupational sensitization in adults, in creative or do‐it‐yourself activities such as decorative craft kits, cosplay costumes, or jewellery making, but children and adolescents may also be affected [7, 8, 9].

Clinical manifestations may widely vary and include acute severe eczema, airborne contact dermatitis, chronic hand eczema, nail manifestations, or paresthesia [10]. It is important to note that, as in our patient, the occurrence of facial eczema may be related to airborne exposure, but also by indirect contact through contaminated hands, which may go unnoticed.

In contrast to occupational environments, where preventive measures and risk information are usually implemented, domestic users are often unaware of the sensitizing and irritant properties of epoxy resins.

Improved consumer information, clearer labelling of sensitization risks and recommendations regarding appropriate protective measures may help reduce the risk of severe ACD associated with recreational use of epoxy resins.

## Author Contributions

**Olivier Dereure:** project administration, writing – review and editing, resources, supervision. **Nadia Raison‐Peyron:** conceptualization, investigation, visualization, writing – review and editing, project administration, supervision, methodology. **Eva Ibeka:** writing – original draft, conceptualization, visualization, writing – review and editing.

## Consent

Written informed consent was obtained from the patient for the Figure 1.

## Conflicts of Interest

The authors declare no conflicts of interest.
